# 原发性肺癌胃肠道转移2例报道并文献复习

**DOI:** 10.3779/j.issn.1009-3419.2011.03.23

**Published:** 2011-03-20

**Authors:** 玉艳 王, 彤同 安, 鹭 杨, 志杰 王, 明磊 卓, 建春 段, 洁 王, 梅娜 吴

**Affiliations:** 100142 北京，北京肿瘤医院，北京大学肿瘤学院胸部肿瘤内科 Department of Thoracic Oncology, Beijing cancer hospital, Beijing University Oncology College, Beijing 100142, China

**Keywords:** 肺肿瘤, 消化道转移, 免疫组化, Lung neoplasms, Gastrointestinal metastasis, Immunohistochemistry

## Abstract

原发性肺癌最常见的远处转移部位包括肺、肝、脑、骨、肾上腺等，胃肠道转移鲜有报道。北京大学肿瘤学院胸部肿瘤内科近期先后收治了经病理证实为原发性肺癌发生胃肠道转移的2例患者，本文将就其诊治及随访情况进行报告，并回顾国内外相关文献，以加深对肺癌少见部位转移的认识，避免漏诊和误诊。

## Abstract (en)

Gastrointestinal metastasis unusually occurs in primary lung cancer instead of lung, liver, brain, bone and adrenal. Recently, we has treated two patients with primary lung cancer metastasized to gastrointestinal system consequently. This paper will conduct the diagnosis, treatment and follow-up report and review related literature in order to deepen the understanding of rarely metastases of lung cancer to avoid misdiagnosis and missed diagnosis.

原发性肺癌转移到胃肠道并不常见，文献报道的发生率差异很大，从10%-14%^[1, 2]^至0.5%-2%^[3, 4]^不等，这一差异主要与检查手段（包括内镜、外科手术和尸检）不同有关。原发性肺癌的胃肠道转移为血行播散，其发生提示疾病晚期且预后不佳。一般分为症状性或无症状性，大部分在原发性肺癌确诊后发现，以消化道转移症状为首发表现的肺癌极少见，诊断难度大，仅偶见文献^[5]^报道。由于临床上缺乏特异性的影像学及内镜下表现^[6, 7]^，已诊断原发性肺癌的患者一旦出现腹部症状应高度怀疑是否存在胃肠道转移，并进行病理检查证实。本文报道的2例患者均发生于原发性肺癌确诊之后，在治疗后复查时发现，以下将进行详细报告。

## 1 病例1

患者，男，71岁，主因“发现右腋下肿物1年余，咳嗽、咳血9个月”于2010年2月入院。入院前1年余自行触摸及右腋下肿物，9个月前出现咳嗽、痰中带血，胸部CT提示：右上肺周围型肿物，伴右侧肺门、纵隔淋巴结肿大，右腋窝淋巴结肿大。2010年1月7日行右腋窝淋巴结穿刺活检，病理示：低分化腺癌浸润。免疫组化结果：CK-7（+），CK 20（-），Napsin A（+），TTF-1（+），符合原发性肺腺癌转移。完善分期检查，明确诊断：右肺上叶周围型低分化腺癌Ⅳ期T3N2M1侵及胸壁右侧肺门、纵隔淋巴结转移右腋窝淋巴结转移。既往：慢性支气管炎病史10余年，吸烟史40年，20支/天，弟弟患“肺癌”。于2010年1月21日开始行一线吉西他滨+卡铂序贯口服特罗凯治疗3周期（入组FAST-ACT2临床试验），2周期评效稳定（stable disease, SD），3周期确认疗效复查胸部CT示右肺内新发结节，评效进展（progressive disease, PD）。2010年4月换二线多西他赛化疗2周期，复查评效：CEA升高，胸部CT提示肺内病变SD，腹部CT提示“胃体后壁结节25 mm×17 mm，凸向胃腔”（图 1A），同时查便潜血阳性。无明显消化道症状，于2010年6月行胃镜检查病理示：（胃底粘膜）可见中低分化腺癌浸润（图 1B），免疫组化结果：CK-7（+），CK20（-），Napsin A（+），TTF-1（+），符合原发性肺癌转移，考虑疾病进展。2010年6月换三线培美曲塞化疗6周期，2、4、6周期评效均为SD（肺内、腋下淋巴结、腹腔淋巴结），患者停止治疗，定期复查随访。目前一般情况良好，ECOG 1级。

**1:**
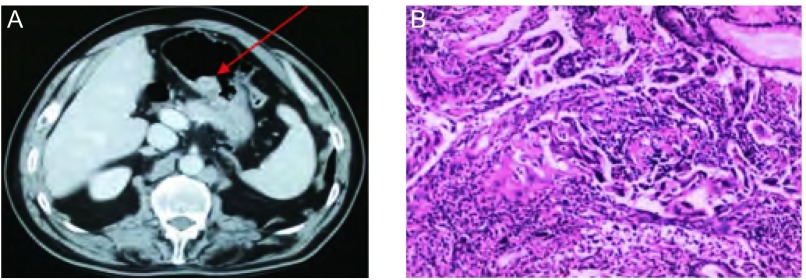
病例1腹部CT及胃镜胃黏膜活检病理表现。A：腹部增强CT示胃体后壁结节（箭头所示）；B：胃镜下胃黏膜活检示低分化腺癌。 Abdomen CT scan and gastroscopic biopsy of case 1. A: Abdomen CT scan showed a nodular in gastric posterial wall; B: Mucosal biopsy by gastroscopy showed low differentiated adenocarcinoma.

## 2 病例2

患者，男，52岁，主因“间断咳嗽咳血1年余”于2010年4月入院。入院前1年余无明显诱因出现咳嗽、咳血，外院行气管镜病理及分期检查，明确诊断为：左肺中心型中分化鳞癌IIIb T4N2M0侵及左肺动脉左肺门、纵隔淋巴结转移。既往2型糖尿病史4年余，吸烟史30年，20支/天，已戒1年，父死于“肺癌”，哥哥患“喉癌”。于2009年8月10日始行同步放化疗，放疗总剂量GTV 70 Gy/35f，同步化疗方案为多西他赛+顺铂2周期，评效为SD，未再治疗。2010年4月22日出现肿瘤标记物（NSE、CYFR A21-1、SCC）升高，复查PET-CT发现“左肾实质新发不规则高代谢肿块，左上腹小肠壁高代谢病灶”，腹部CT示“小肠肠壁增厚并肠系膜肿物，左肾弥漫浸润性占位”（图 2A）。于2010年5月6日左肾穿刺活检病理示“中分化鳞癌”。2010年5月26日行腹腔镜下腹腔肿物活检病理示“纤维组织中可见中分化鳞癌细胞浸润，符合肺癌转移（图 2B）”。2010年6月始行二线吉西他滨化疗2周期，评效为PD。患者一般情况较差，ECOG 2级，进食差，腹痛明显，建议换厄洛替尼治疗，但患者放弃进一步抗肿瘤治疗，2011年2月13日死于肿瘤进展导致的呼吸衰竭。

**2:**
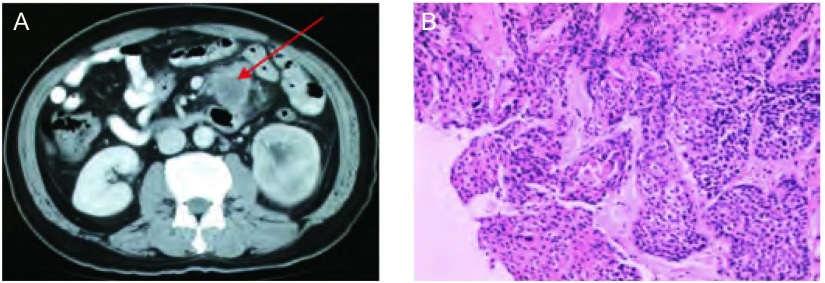
病例2腹部CT及腹腔镜肿物活检病理表现。A：腹部增强CT示小肠肠壁增厚并肠系膜肿物（箭头所示）；B：腹腔镜腹腔肿物活检示中分化鳞癌。 Abdomen CT scan and laparoscopic mass biopsy of case 2. A: Abdomen CT scan showed thickening small intestinal wall and mesenteric mass; B: Laparoscopic mass biopsy showed mid-differentiated squemous cell carcinoma.

## 3 讨论

1942年Oschner和DeBakey首先报告了3, 047例原发性肺癌患者的尸检标本，胃肠道受累131例，总发生率为4.3%^[8]^。继之，1982年Cancer上发表了一项研究^[9]^，回顾了423例原发性肺癌患者的尸检资料，发现胃肠道转移者58例（14%），食管是最常见的受累部位（33例），中段发生最多（16例，49%），其中14例为肿瘤直接侵犯。1996年日本Saitama肿瘤中心^[4]^通过手术或解剖证实，1, 635例原发性肺癌消化道（食管除外）转移者30例（7例生前诊断，23例死后确诊），总体发生率为1.8%，不同部位转移发生率为小肠1.1%，胃0.4%，结肠0.5%。同样，2007年意大利的研究也发现胃肠道转移中小肠是最常见的转移部位，占67%（12/18），其次为胃占22%（4/18），大肠最少为11%（2/18）^[5]^。2009年韩国的研究也通过CT检查发现肺癌常见的胃肠道转移部位依次为小肠占84%（*n*=26），胃占10%（*n*=3）和结肠占6%（*n*=2）^[3]^。

从大体形态上来说，原发性肺癌转移到消化道常表现为肠道多灶性受累，缺乏组织学过渡区。在组织学上，其形态与原发肿瘤一致，并具有相应的特异性免疫组化表现，如TTF-1、CK7阳性，而CDX2、CK20阴性^[10]^。不同组织类型转移到胃肠道的原发性肺癌中，既往认为最常发生的组织类型为鳞状细胞癌33%（19/58），其次为大细胞癌29%（17/58）和燕麦细胞癌19%（11/58）^[9]^，但近期文献报告分化差的大细胞未分化癌和腺癌更为常见，日本1996年的研究^[4]^发现大细胞癌转移最多占3.7%（11/30），其次为腺癌占2.3%（7/30）和小细胞癌占1.7%（5/30），鳞癌最少为0.7%（2/30）。2007年Rossi等^[5]^报告的以消化道症状为首发表现的回顾性研究总结了18例通过活检证实为原发性肺癌转移到胃肠道的患者资料，所有标本均进行了TTF-1、CDX2、CK7、CK 20等标志物的免疫组化染色。10例为大细胞未分化癌（56%），8例为腺癌（44%）。免疫组化CK7阳性率为100%（18/18），TTF-1阳性率为89%（16/18）；2例TTF-1阴性的患者均为大细胞未分化癌；CK20和CDX2均为阴性。

大部分原发性肺癌患者发生胃肠道转移时无症状，有症状的患者最常见的表现为吞咽困难，特别是胃肠道近端（食管、胃）受累，累及到远端胃肠道（小肠、大肠）时最常见表现为疼痛^[9]^。Hillenbrand等^[2]^的报道发现58例原发性肺癌肠道转移者最常见的临床表现为穿孔34例（59%），其次为梗阻17例（29%）和出血6例（10%），无症状者1例（2%）。肠套叠不常见，仅印度有1例个案报告^[11]^。韩国的回顾性研究中发现31例患者中肠套叠7例（23%）、肠穿孔6例（19%）和肠梗阻4例（13%），无症状者14例（45%）^[3]^。

就辅助检查而言，CT是较为常用的检查方法。2009年韩国的研究中31例肺癌胃肠道转移患者CT表现分别为管壁增厚（*n*=14）、腔内息肉样肿物（*n*=14）和外生型肿物（*n*=3）。这些病灶密度不一，等密度者19例，低密度者7例，高密度者5例。便潜血试验也是有效的辅助检查手段，进行性腹痛且便潜血持续阳性时消化道转移的发生率较高，而确诊仍要靠内窥镜或手术活检^[3]^。

我国1984年-2010年间先后有4篇相关文献^[12-15]^共报告了8例原发性肺癌发生小肠转移的病例，其中肺腺癌2例，小细胞癌1例，鳞状细胞癌3例，大细胞未分化癌2例。患者均为男性，平均年龄70岁，大部分合并肠梗阻。

肺癌患者胃肠道转移预后极差。韩国2009年的报道胃肠道转移确诊后中位生存期为96.5 d，其中1例患者进行胃肠道转移病灶姑息切除术及原发肺癌根治术，并给予术后化疗存活5年未复发，提示在严格选择的患者中手术是具有潜在治愈可能的手段^[3]^。Rossi等^[5]^报告的14例患者大部分在短期内死亡，中位随访时间为3个月（1个月-14个月），其中2例发生多部位转移后仍存活；2例消化道为唯一转移部位，接受了肺叶切除术和化疗后长期存活且无相关症状。日本报道出现消化道症状后患者中位生存期仅为49 d，5例患者消化道转移为直接死因^[4]^。我国报告的几例小肠转移患者平均生存期仅为4周-8周^[12-15]^，随访情况为：术后存活1个月、2个月、4个月的患者分别为1例、2例和1例；1例术后存活超过2年，3例生存期不详。

本研究中2例患者均为无症状性，均经病理证实，免疫组化结果与原发肺癌一致，治疗上予全身化疗和局部对症治疗，1例肿瘤控制稳定，1例确诊后化疗无效进展死亡。

总之，尽管肺癌胃肠道转移与远期预后不良有关，但转移灶姑息切除+肺原发肿瘤切除术联合化疗可能使某些孤立性胃肠道转移患者获得较好的疗效和较长生存。对临床和病理科医生而言，结合本研究并文献复习，我们提出以下建议：（1）在诊断胃肠道恶性肿瘤时需警惕肺转移性癌的可能；（2）形态学上不常见的胃肠道未分化癌，提示可能为转移性癌，建议利用胃肠道和肺癌常见的特异性标志物进行免疫组化染色鉴别，包括TTF-1、CDX2、CK7和CK20等；（3）肺癌胃肠道转移为弥漫性血行转移，提示疾病晚期，但全面分期检查显示为胃肠道单脏器孤立性转移病灶，且原发肺癌（非小细胞肺癌）系可完全性手术切除的患者（T1, 2N0, 1M0），可考虑行胃肠道转移病灶和肺原发病灶的手术切除，随后全身化疗可能改善患者的预后、延长生存期，对此尚需进一步研究证实。
